# The Influence of Cell Isolation and Culturing on Natriuretic Peptide Receptors in Aortic Vascular Smooth Muscle Cells

**DOI:** 10.3390/cells14010051

**Published:** 2025-01-04

**Authors:** Christine Rager, Tobias Klöpper, Sabine Tasch, Michael Raymond Whittaker, Betty Exintaris, Andrea Mietens, Ralf Middendorff

**Affiliations:** 1 Institute of Anatomy & Cell Biology, Faculty of Medicine, Justus-Liebig-University, Aulweg 123, 35392 Giessen, Germany; christine.rager@monash.edu (C.R.);; 2 Drug Delivery, Disposition, and Dynamics (D4), Monash Institute of Pharmacy & Pharmaceutical Sciences (MIPS), Monash University, 381 Royal Parade, Parkville, VIC 3052, Australia; michael.whittaker@monash.edu; 3 Pharmacy and Pharmaceutical Sciences Education, Monash Institute of Pharmacy & Pharmaceutical Sciences (MIPS), Monash University, 381 Royal Parade, Parkville, VIC 3052, Australia

**Keywords:** vascular smooth muscle cells, aorta, cGMP signaling, natriuretic peptides, nitric oxide, phenotype switch, biological sex

## Abstract

Vascular smooth muscle cell (SMC) relaxation by guanylyl cyclases (GCs) and cGMP is mediated by NO and its receptor soluble GC (sGC) or natriuretic peptides (NPs) ANP/BNP and CNP with the receptors GC-A and GC-B, respectively. It is commonly accepted that cultured SMCs differ from those in intact vessels. Nevertheless, cell culture often remains the first step for signaling investigations and drug testing. Previously, we showed that even popular reference genes changed dramatically after SMC isolation from aorta. Regarding NP receptors, a substantial amount of data relies on cell culture. We hypothesize that the NP/cGMP system in intact aortic tunica media differs from isolated and cultured aortic SMCs. Therefore, we studied isolation and culturing effects on the expression of NP receptors GC-A, GC-B, and NP clearance receptor (NPRC) compared to sGC. We investigated intact tunica media and primary SMCs from the longitudinal halves of the same rat aorta. GC activity was monitored by cyclic guanosine monophosphate (cGMP). In addition, we hypothesize that there are sex-dependent differences in the NP/cGMP cascade in both intact tissue and cultured cells. We, therefore, analyzed a male and female cohort. Expression was quantified by RT-qPCR comparing aortic media and SMCs with our recently validated reference gene (RG) small nuclear ribonucleoprotein 2 (U2). Only GC-A was stably expressed. In intact media, GC-A exceeded GC-B and NPRC. However, GC-B, NPRC, and sGC were dramatically upregulated in cultured SMCs of the same aortae different from the stable GC-A. The expression was mirrored by NP-induced GC activity. In cultured cells, changes in GC activity were delayed compared to receptor expression. Minor differences between both sexes could also be revealed. Thus, isolation and culture fundamentally alter the cGMP system in vascular SMCs with potential impact on drug testing and scRNAseq. Especially, the dramatic increase in the clearance receptor NPRC in culture might distort all physiological ANP, BNP, and CNP effects.

## 1. Introduction

Vascular smooth muscle cells (SMCs) account for the majority of cells in the walls of arterial blood vessels [1,2]. While the importance of vascular SMCs for the regulation of resistance arteries is accepted, their relevance in elastic arteries (e.g., aorta) is still underestimated [3,4]. Their contraction and relaxation are precisely regulated by different pathways to adapt vessel diameter and consequently fine-tune blood pressure [5]. The second messenger molecule cyclic guanosine monophosphate (cGMP) is a key player in SMC relaxation [6,7]. Intracellular cGMP production is induced in two different ways. cGMP production by the cytosolic receptor soluble guanylyl cyclase (sGC) in response to nitric oxide (NO) is commonly known [8,9,10]. In addition, the membrane-bound guanylyl cyclases-A (GC-A) and -B (GC-B) generate cGMP when binding their specific ligands, the natriuretic peptides (NPs) [11]. ANP and BNP bind to GC-A while CNP activates GC-B. A third NP receptor, the NP clearance receptor (NPRC), binds the NPs with comparable affinity [12]. The NPRC is thought to be mainly responsible for NP clearance; however, additional functions are suggested [13,14].

With cGMP being a key mediator of vasodilation, this signaling cascade is targeted by several cardiovascular therapeutics. While nitrates, sGC stimulators, and activators enhance sGC-dependent cGMP generation, phosphodiesterase inhibitors interfere with its degradation. NP-dependent signals are currently explored in cardiovascular disease. The BNP precursor NT-pro-BNP is an established biomarker for heart failure and NP-based therapeutics are being developed. The availability of NPs to bind to their signaling receptors GC-A and GC-B underlies additional local control. While NPRC competes for the NP ligand, the metalloproteinase neprilysin (NEP) degrades NPs. The latter one is also targeted by the NEP inhibitor sacubitril in the treatment of heart failure. In view of the different options to influence cGMP signals, it is of utmost importance to understand the intricate interplay of NPs with the different signaling components that shape the cGMP signal and illustrates an urgent need for establishing reliable models.

Vascular SMCs have been and are still intensively studied in cell culture experiments. Although cell culture essentially contributes to the basic understanding of intracellular signaling, it has been shown that in vitro experiments reflect physiological circumstances to a limited extent, and we hypothesize that the NP/cGMP cascade is no exception. Yet, systematic investigations comparing the cGMP system in intact tissue and corresponding cells are still scarce. In a previous study [15], we evaluated the suitability of reference/housekeeping genes (RGs) for the comparison between intact aortic media and derived primary aortic SMCs by quantitative PCR (RT-qPCR). Besides the identification of U2 as a valid RG, we found that most RGs, especially classic examples such as beta-actin, did not fulfill the criteria for this comparison and showed a significantly different expression between the cells and the tissue. In our present study, we now aim to evaluate to what extent the expression of the cGMP signaling cascade changes when aortic SMCs are isolated and cultured.

The interest in the development of sex-specific medicine is increasing [16]. This inevitably requires researchers to include the biological sex as a limiting factor for their studies. It has been shown that sex hormones can have a substantial impact on cardiovascular physiology [17,18,19]. However, knowledge of the cardiovascular actions of NPs and their therapeutic potential in both sexes is mostly focused on the heart, while there is little information on aortic tissue [20,21]. Since we hypothesize that the NP/cGMP cascade differs between both sexes, we decided to additionally include a male and a female study cohort to explore sex-related differences in the cGMP signaling cascade.

## 2. Materials and Methods

### 2.1. Animals

Tissue was obtained from 2- to 3-month-old male and female Wistar rats (*Rattus norvegicus*) housed in the animal facility of the Veterinary Faculty at Justus-Liebig-University (JLU) Giessen, Germany. All the procedures were conducted according to the guidelines of the German Animal Welfare Act and approved by the Committee for Laboratory Animals of the JLU Giessen, case number JLU Nr. 577_M (approved period: January 2020–December 2022). The rats were anesthetized with CO_2_ and sacrificed by cervical dislocation.

### 2.2. Tissue Preparation

Aortae were dissected, transferred into MEM 1X medium (Gibco, Thermo Fisher Scientific, Waltham, MA, USA), supplemented with 1% (*v*/*v*) Penicillin–Streptomycin (Pen/Strep, 10,000 U/mL, Sigma Aldrich, St. Louis, MO, USA), and stored on ice. The SMC layer (media) was prepared in cold MEM 1× + Pen/Strep. After removing the outer fat, branching vessels were cut, and the media was opened longitudinally from the aortic arch until the thoracic bifurcation. Onehalf of the same aorta was used for primary SMC extraction (see following chapter), while the second half was prepared as follows: Starting from the proximal end, the surrounding connective tissue (adventitia) was manually removed using curved tweezers. The endothelium (intima) was carefully scratched away. For RNA isolation, the remaining moisture was dried off the tissue, which was subsequently scaled, and immediately frozen in liquid nitrogen.

### 2.3. SMC Extraction and—Culture

The extraction of the SMCs from the aortic media required a two-step enzymatic digestion with collagenase (Collagenase Type II, Sigma-Aldrich, St. Louis, MI, USA). The aorta was prepared as described above and the longitudinal half designated for SMC extraction was first digested with collagenase (10% (*w*/*v*) in MEM 1× + Pen/Strep) at 37 °C for 15 min. After the transfer into fresh MEM 1× + Pen/Strep, the tissue was manually separated from the adventitia starting at the proximal end. The thin intimal layer was scratched off and the remaining media was cut into small pieces using sterile scissors. The media pieces were transferred into fresh MEM 1× + Pen/Strep with collagenase (10% (*w*/*v*)) and incubated for 30 min at 37 °C. Afterward, digestion was stopped by dilution with approximately 5 mL prewarmed cell culture medium DMEM/F-12 (Gibco, Thermo Fisher Scientific, Waltham, MA, USA) + Pen/Strep containing 10% (*v*/*v*) fetal bovine serum (FBS, by Gibco, Thermo Fisher Scientific, Waltham, MA, USA). The suspension was strained through a cell strainer (40 µM grid size by BD Biosciences, Bedford, MA, USA) under sterile conditions, and the flow-through, containing the singularized SMCs, was centrifuged for 10 min at 1500 rpm and room temperature. The cell pellet was resuspended in 5 mL fresh pre-warmed DMEM/F-12 + FBS + Pen-Strep and transferred into 25 cm^2^ sterile plastic cell culture flasks (Thermo Fisher Scientific, Waltham, MA, USA). The SMCs were cultured at 37 °C, 5 % (*v*/*v*) CO_2_ and media was changed every 2 to 3 days. The extraction passage was cultured for 7 days before the SMCs were trypsinized (Trypsin-EDTA solution, Sigma-Aldrich) for 5 min at 37 °C after washing (DPBS, Gibco, Thermo Fisher Scientific, Waltham, MA, USA) and transferred into fresh culture flasks. Cells (P1 till P3 after extraction) were seeded as follows: 4 to 5 × 10^5^ cells per 25 cm^2^ flask for the RNA extraction and follow-up RT-qPCR, and 4 × 10^4^ cells per well in 48-well plates for the cGMP ELISA.

### 2.4. RNA Extraction

Total RNAs from the isolated and cultured SMCs and from the intact media of the same animal were prepared using the RNeasy^®^ Mini Kit (Qiagen, Hilden, Germany). Isolation was performed according to the manufacturer’s protocols using the spin technology with on-column DNase digestion (RNase-Free DNase Set (50), Qiagen, Hilden, Germany). For isolation from intact tissue, approximately 10 to 20 mg of rat aortae (male and female, each n = 12) were frozen in liquid nitrogen and pulverized using a hammer. RLT buffer with 1 % (*v*/*v*) 2-Mercaptoethanol (Roth, Karlsruhe, Germany) and a sterile metal bead were added, and the sample was processed for 3 min at 300 Hz in a tissue lyser mixer mill MM400 (Retsch, Haan, Germany). The lysate was centrifuged (5 min at 12,000 rpm) and the supernatant was further processed according to the manufacturer’s instructions. The final RNA concentration was determined using a NanoDrop^TM^ 2000/2000c spectrophotometer (Thermo Fisher Scientific, Waltham, MA, USA). The RNA samples were stored at −80 °C.

### 2.5. cDNA Synthesis

A total of 2 µg of the extracted RNA served as the template for the cDNA synthesis. SuperScript^TM^ II Reverse Transcriptase and Oligo(dT)_20_ primer (both from Invitrogen, Thermo Fisher Scientific, Waltham, MA, USA) were applied according to the manufacturer’s instructions using the Mastercycler gradient 5331 (Eppendorf, Hamburg, Germany). The consistency of the reverse transcription (RT) was verified by the measurement of the cDNA concentration. In case of large differences, cDNAs were diluted to the same concentration for the subsequent RT-qPCR measurement.

### 2.6. RT-qPCR

RT-qPCR was performed using the iCycler IQ PCR system and the iQTM SYBR^®^ Green Supermix (both from Bio-Rad, Hercules, CA, USA) using the cycler steps listed in Table 1 below. For the quality control of the RT-qPCR, primers and -RT controls were applied right next to the PCR products on a 3% agarose (*w*/*v*) ethidium bromide gel). The length of the double-stranded PCR products was checked after visualization in a UV chamber (Gel Jet Imager running the software Intas GDS Touch 2, Intas Science, Göttingen, Germany).

Custom DNA-Oligos (0.01 µM, salt-free, lyophilized, MALDI-TOF MS quality analysis) were purchased from Eurofins, Hamburg, Germany. Primer details for the RG and the genes of interest are listed below. The efficiency (*E*) of each primer pair was calculated based on a cDNA dilution series with three steps (undiluted, 1:2, and 1:4). Every dilution sample was measured as a triplet in a usual RT-qPCR run according to the qPCR steps given in Table 1. The resulting Ct mean values of each dilution level were plotted against the concentration of the diluted sample (log10 scale) and used to create a descending slope (*m*) by linear regression according to the following calculation:

*E* = 10^[−1/*m*]^ − 1

Under the ideal conditions of the PCR and 100% efficiency of the primers, a duplication of the nucleic template sequence can be expected. We only used primer sets that achieved an efficiency between 100–120%.

**Small nuclear RNA—U2:**

Forward 5′-ATCTGATACGTCCTCTATCC-3′

Reverse 5′-GTGGACGGAGCAAGCTCCTA-3′

Amplicon size: 83 bp

Efficiency: 102% [22]

**Guanylyl cyclase A—GC-A:**

Forward 5′-GACCTACTGCCCTGCTGTTC-3’

Reverse 5’-GAGAGGTGGAAGCTGGACAC-3’

Amplicon size: 77 bp

Efficiency: 102% [23]

**Guanylyl cyclase B—GC-B:**

Forward 5’-TGGCTCTTAGGAGAGCGAAA-3’

Reverse 5′-GCCCATCCAAGTACCAACAG-3′

Amplicon size: 84 bp

Efficiency: 119% [23]

**Natriuretic peptide clearance receptor—NPRC:**

Forward 5′-CTCCTTGCAAATCATGTGGCCT-3′

Reverse 5′-TCTTGGTGATTTCGCCTCTCA-3′

Amplicon size: 142 bp

Efficiency: 120%

(BLAST: NM_012868.1, (https://www.ncbi.nlm.nih.gov/tools/primer-blast/), accessed on 18 September 2022))

**Soluble Guanylyl cyclase subunit beta 1—sGC:**

Forward 5′-GCGGACACCATGTACGGTT-3′

Reverse 5′-GCAGCCACCAAGTCATAGGT-3′

Amplicon size: 170 bp

Efficiency: 115%

(BLAST, NM_012769.2; (https://www.ncbi.nlm.nih.gov/tools/primer-blast/), accessed on 18 September 2022)

The relative mRNA amount of the target genes was calculated using the ΔCt method by normalization to the RG U2 [15].

### 2.7. cGMP ELISA

cGMP levels after the NP treatments were determined by ELISA (Institute for Hormone and Fertility Research [IHF], Hamburg, Germany) based on a previously described assay [24,25] following the principles of a competitive ELISA. Therefore, Immuno 96-well plates (Thermo Fisher Scientific, Waltham, MA, USA) were coated with goat-anti-rabbit IgG (H + L) antibody solution (Invitrogen, Thermo Fisher Scientific, Waltham, MA, USA). Sodium chloride, Tween, Hydrogen peroxide (H_2_O_2_), and Tetramethylbenzidine (TMB) were obtained from Roth. Sodium acetate was obtained from Merck, Darmstadt, Germany, and citric acid from Sigma-Aldrich. cGMP standard (Biolog, Hayward, CA, USA) dilution series was prepared in E-PBS [0.1 M sodium triphosphate, 0.15 M NaCl, 5 mM EDTA, 0.2 % (*w*/*v*) BSA, 0.01 % (*w*/*v*) Thimerosal; (pH 7.0)].

For the cGMP ELISA, the SMCs were cultured in 48-well plates until 80% of the well bottom was covered. The cell culture media was exchanged with FBS-free MEM 1× + Pen-Strep for at least 4 h for equilibration. For treatment, MEM 1× + Pen-Strep was prewarmed and 3-Isobutyl-1-methylxanthine (IBMX, 0.25 nM, Sigma-Aldrich, St. Louis, MI, USA), a phosphodiesterase inhibitor dissolved in Dimethyl sulfoxide (DMSO, Roth, Karlsruhe, Germany), and ANP or CNP (each 100 nM, Bachem, Bubendorf, Switzerland) were added. The same medium with IBXM only was applied as the control medium. Treatment at 37 °C and 5% (*v*/*v*) CO_2_ was stopped after 30 min by the addition of ice-cold 100% (*v*/*v*) ethanol and immediately freeze down at −20 °C for at least 1 h. The cells and supernatant were detached and centrifuged for 30 min at 4 °C and 20,000× *g*. Clear supernatant was transferred into a fresh tube and lyophilized. The remaining pellet was resuspended in 140 µL E-PBS.

Longitudinal halves of one aorta, prepared as mentioned above (see Tissue preparation), were scaled and equilibrated in prewarmed MEM 1× + Pen-Strep for 10 min at 37 °C. For treatment, the tissues were incubated with MEM 1× + Pen-Strep containing IBMX (0.25 nM) and ANP or CNP, each at a concentration of 100 nM for 30 min at 37 °C. One longitudinal half was treated with ANP, and the other half of the same vessel was treated with CNP. The supernatant was frozen immediately in liquid nitrogen and stored at −80 °C until measurement as follows.

The samples and standards (50 µL per well) were loaded in duplicates on the antibody-coated 96-well plates together with 100 µL cGMP antiserum [1:80,000] and 50 µL Biotin-cGMP tracer [170 fM] were added according to manufacturer’s suggestions. The plates were incubated for at least 18 h in a dark, wet chamber at 4 °C. The solution was removed from wells and 200 µL of HPR-Streptavidin solution [0.152 µg/mL in E-PBS] (Vector Laboratories, San Francisco Bay Area, CA, USA) was added per well. The plate was incubated for 30 min in a dark, wet chamber at 4 °C. The wells were washed 3 times with washing buffer [0.5% NaCl (*w*/*v*); 0.02% Tween 20 (*v*/*v*)], and 250 µL HPR-substrate solution [0.01 M sodium acetate; 0.0045 M citric acid; 0.0038% H_2_O_2_ (*v*/*v*) and 0.001 % TMB (*w*/*v*)] was added per well. After an incubation for 40 min in a dark, wet chamber at room temperature, 50 µL H_2_SO_4_ 2M (Roth) was added. The emission intensity was detected photometrically by a DIAS microplate reader (Dynatech Laboratories, Denkendorf, Germany) at 450 nm. Based on the blanks and the standard, the concentration of cGMP in the samples was calculated automatically by the software Revelation 2.0 (Dynatech Laboratories). For the tissue, additionally, the cGMP concentration was normalized to the tissue weight.

### 2.8. Statistical Analysis

For all the underlying statistical analyses, GraphPad Prism version 10.2.2 (397) for Windows (GraphPad Software, La Jolla, CA, USA, www.graphpad.com, last accessed for analysis: 15 April 2024) was used. All data were pre-checked for normal distribution. The n-number (given in each figure) refers to the number of biological replicates (number of animals). Detailed descriptions of the statistical evaluation and significant differences are given in the corresponding figure legends. Data are depicted as the median ± interquartile range.

## 3. Results

### 3.1. Culturing Aortic SMCs Dramatically Changes the Expression of Gc-b, sGC, and Nprc but Not of Gc-a in Comparison to the Intact Media

Since NP/cGMP signaling is one of the most relevant pathways for SMC relaxation, we decided to first compare the gene expression levels of the cGMP-generating *Gc-a* and *Gc-b*, as well as the NP-clearing receptor *Nprc* in the intact media of the rat aorta and in cultured aortic SMCs using RT-qPCR.

**Figure 1:**
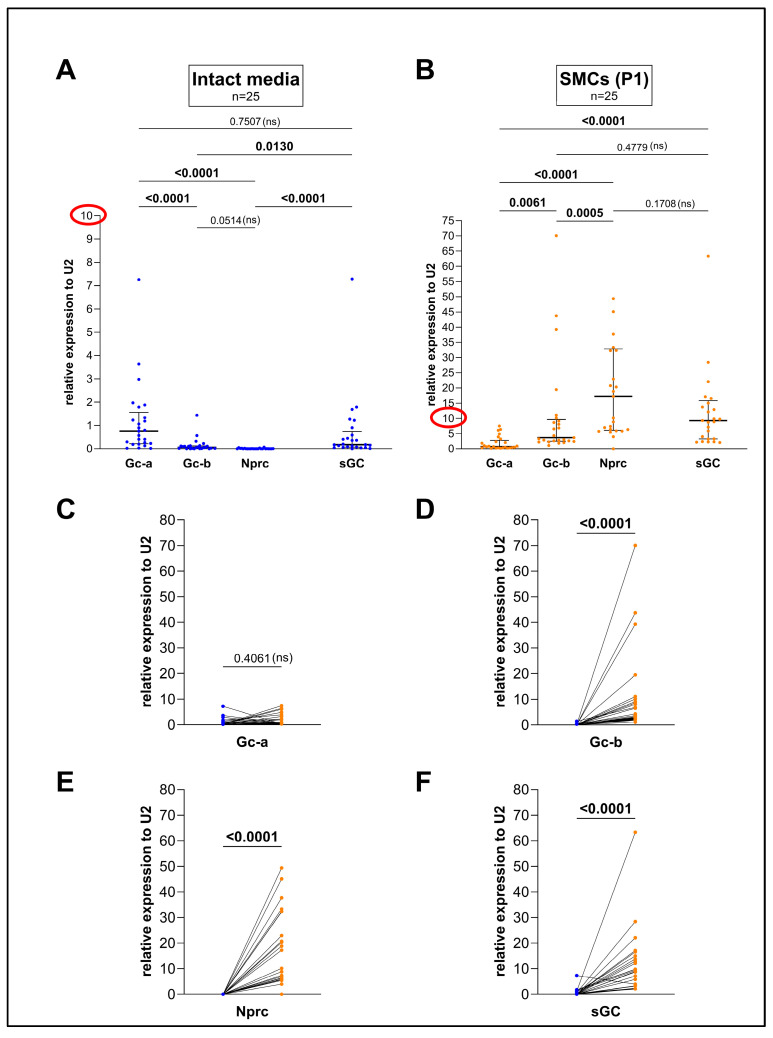
Relative gene expression analyses of the natriuretic peptide (NP)/cyclic guanosine monophosphate (cGMP) pathway components in the intact media and corresponding cultured smooth muscle cells (SMCs) of the rat aortae. The gene expression levels of guanylyl cyclase A (*Gc-a*) and -B (*Gc-b*), natriuretic peptide clearance receptor (*Nprc*), and soluble guanylyl cyclase (*sGC*) in the (**A**) intact media (blue dots) and (**B**) corresponding cultured aortic SMCs of the first culture passage after extraction (P1) (orange dots). Please note the different scales of the *y*-axis in both graphs highlighted by the red circles. Differences in (**A**, **B**) were analyzed by non-parametric Friedman test with Dunn’s correction for multiple comparisons of paired measurements. Individual values are depicted for each gene. The median is indicated by a thick line while whiskers indicate the interquartile range. Direct comparisons between intact media (blue dots) and corresponding cells (orange dots) of each individuum are visualized by connecting lines (**C**–**F**)). Individual data points visualize the dispersion and the slope of each line and the amount of change in gene expression for each individual gene. Differences were analyzed using a non-parametric Wilcoxon matched-pairs signed rank test. All data were normalized to small nuclear ribonucleoprotein *U2* according to the ΔCt method for relative expression analysis. The total cohort (n = 25) consists of male (n = 13) and female (n = 12) rats. Numeric *p*-values are given for each comparison, while significant differences are highlighted in bold and non-significant values are indicated with (ns).

We found that in intact aortic media, *Gc-a* expression significantly exceeded the expression of *Gc-b* and *Nprc*, which both showed the lowest expression (Figure 1A). As another prominent source of cGMP, we included the intracellular NO receptor sGC in our studies. *sGC* was significantly higher expressed than *Gc-b* and *Nprc*, but no significant difference in comparison to *Gc-a* could be detected in the tissue (Figure 1A).

**Figure 2:**
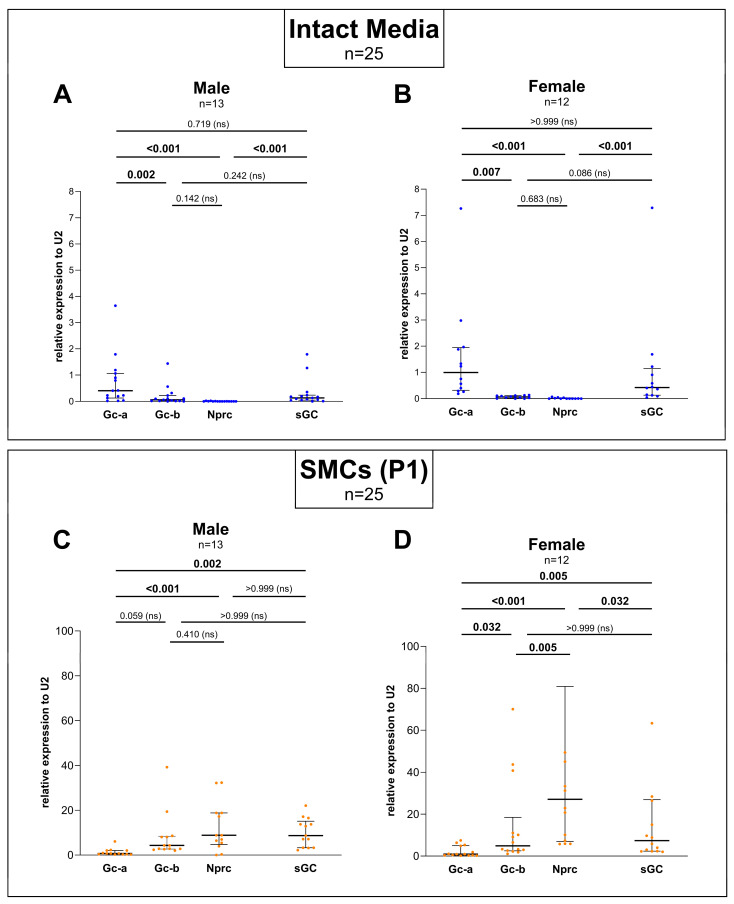

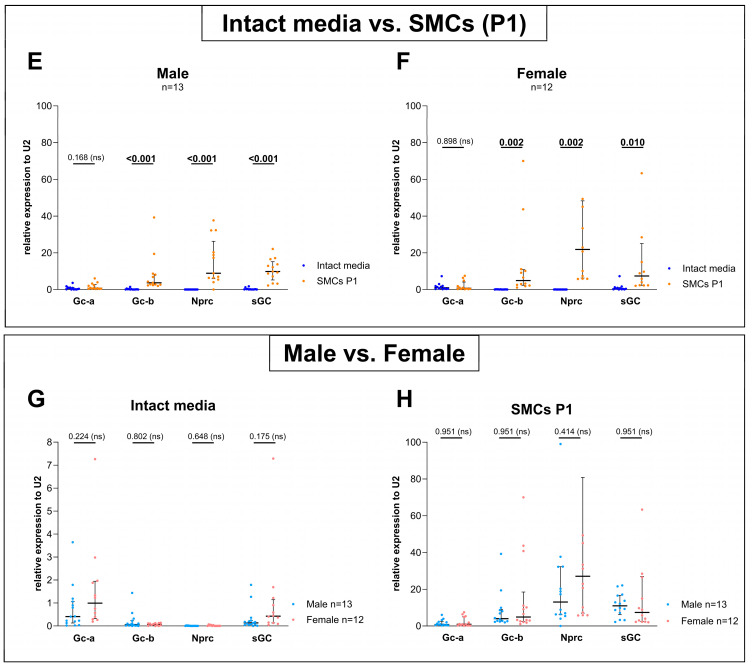
Sex-dependent gene expression analyses of the natriuretic peptide (NP)/cyclic guanosine monophosphate (cGMP) pathway components in the intact media and corresponding cultured smooth muscle cells (SMCs) of the rat aortae. The expression levels of guanylyl cyclase A (*Gc-a*) and -B (*Gc-b*), natriuretic peptide clearance receptor (*Nprc*), and soluble guanylyl cyclase (*sGC*) in the intact media (blue dots) and corresponding cultured aortic SMCs (orange dots) of the first culture passage after extraction (P1) were normalized to small nuclear ribonucleoprotein *U2* according to the ΔCt method for relative gene expression analysis. Differences in the expression of the genes studied in the intact media of (**A**) male (n = 13) and (**B**) female (n = 12) rats and the corresponding SMCs of the same (**C**) male and (**D**) female rats were analyzed by non-parametric Friedman test with Dunn’s correction for multiple comparisons of paired values. Pairwise comparison of the relative gene expression levels between the intact media and corresponding cultured SMCs of (**E**) male and (**F**) female rat aortae was performed by non-parametric Wilcoxon’s *t*-test with Holm–Sidak correction for multiple comparisons of paired measurements. The comparison of expression levels in the (**G**) intact media and (**H**) cultured SMCs between the male (light blue dots) and female rats (pink dots) was performed by a non-parametric Mann–Whitney test with Holm–Sidak correction for multiple comparisons of unpaired measurements. Individual values are depicted for each gene. The median is indicated by a thick line while whiskers indicate the interquartile range. Numeric *p*-values are given for each comparison while significant differences are highlighted in bold and non-significant values are indicated with (ns).

In the isolated and cultured SMCs, a difference in *Gc-b* and *Nprc* expression was detectable in the cells extracted from the female aorta (Figure 2D). Given that this difference was not seen in the male subgroup (Figure 2C), but in the total group (Figure 1B), most likely the spread in the female expression levels accounts for a higher average value causing the significant difference in the total group (Figure 1B).

Visualizing the data pairs of the intact media and cultured SMCs of the same animals with a connecting line (Figure 1C–F), the increase as well as the spread of *Gc-b, Nprc*, and *sGC* expression becomes apparent, especially in the cultured SMCs. Interestingly, *Gc-a* expression remained nearly unchanged in the intact media and the SMCs of the same aortae when performing pairwise comparisons of the individual genes (Figure 1C). In contrast, the expression of *Gc-b* (Figure 1D) and *Nprc* (Figure 1E) increased drastically under culturing conditions, and the same was the case for *sGC*.

### 3.2. Sex-Specific Differences Were Found When Comparing the Expression Between Gc-a, Gc-b, Nprc, and sGC but No Differences Could Be Detected Between Male and Female When Comparing Each Gene Separately

Whereas in Figure 1 the expression data were shown without taking the biological sex into account, Figure 2 displays the male and female cohort separately (Figure 2A–F) as well as the comparison between the gene expression in the two sexes (Figure 2G,H).

In general, the gene expression profile of both cohorts, male and female (Figure 2A–F), mirrors that of the entire group (Figure 1A,B). However, in detailed statistical analyses, subtle differences in the expression between male and female can be observed.

While a significant difference between the expression of *Gc-b* and *sGC* could be detected in the intact media of the total cohort (Figure 1A), this difference was abolished when analyzing the male (Figure 2A) and female (Figure 2B) tissues separately. However, it cannot be excluded that the difference between the sexes escaped detection due to a lower n-number of the male (n = 13) and female (n = 12) animals.

In the isolated and cultured SMCs, a difference in *Gc-b* and *Nprc* expression was detectable in the cells extracted from the female aorta (Figure 2D). Given that this difference was not seen in the male subgroup (Figure 2C), but in the total group (Figure 1B), most likely the spread in the female expression levels accounts for a higher average value causing the significant difference in the total group (Figure 1B).

When comparing the expression of the genes under study in the intact media with the corresponding cultured SMCs for both sexes separately, significant differences were found for *Gc-b, sGC*, and *Nprc*, but not for *Gc-a* (Figure 2E,F). Thus, the expression profile of the male and female groups displays the same expression profile as the total cohort (see Figure 1A,B).

Comparing the two sexes, the statistical analyses for each gene did not show any significant differences neither in the intact media (Figure 2G) nor in the cultured cells (Figure 2H).

### 3.3. cGMP Production by the GC-A Ligand ANP and by the GC-B Ligand CNP in Cultured SMCs Differed in Higher Passages in Comparison to the Intact Media but Not Within Early Passages

**Figure 3:**
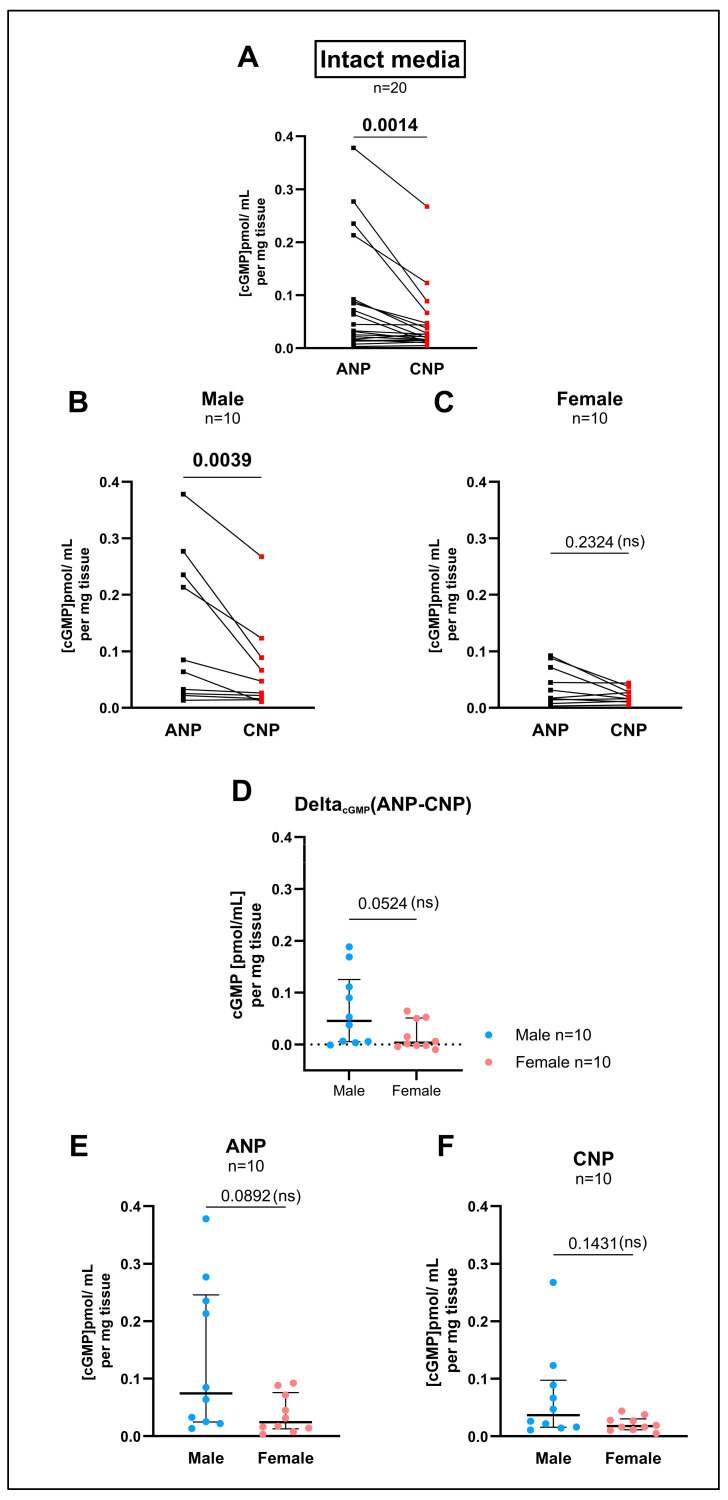
Measurement of cyclic guanosine monophosphate (cGMP) release by the intact media after the natriuretic peptide (NP) treatment. The cGMP concentrations of the sample supernatants, given in pmol/mL, were normalized to the weight (mg) of the aortic tissue samples. cGMP measurement followed a 30 min incubation time with either atrial natriuretic peptide (ANP) or C-type natriuretic peptide (CNP) at a concentration of 100 nm each. The basic cGMP level of the IBMX (phosphodiesterase inhibitor) control treatment was on average 0.51 pmol/mL per mg tissue. (**A**–**C**) Lines connect the single values for the ANP- (black) and CNP- (red) stimulated cGMP of the same individuum. Differences in the ANP- and CNP-induced cGMP release in (**A**) the total study cohort (n = 20), and (**B**) in the male (n = 10) and (**C**) female rats (n = 10) only were calculated by two-tailed non-parametric Wilcoxon’s matched-pairs signed rank test. (**D**) To explore the influence of sex on the cGMP release, the difference between the ANP- and CNP-induced cGMP release was calculated as Delta_cGMP_(ANP-CNP) and compared between the male (light blue dots) and female (pink dots) individuals by non-parametric Mann–Whitney test of unpaired measurements. Additionally, the (**E**) ANP- and (**F**) CNP-induced cGMP release between the male and female rats (n = 10 each) was calculated by the non-parametric Mann–Whitney test of unpaired measurements. Individual values are depicted for each gene. Median is indicated by a thick line while whiskers indicate the interquartile range. Numeric *p*-values are given for each comparison while significant differences are highlighted in bold and non-significant values are indicated with (ns). Due to the reduced n-number in the male and female groups compared to the total cohort, the resulting *p*-values should be interpreted in an explorative manner.

In the intact media, cGMP production in response to ANP or CNP differed significantly in the total cohort (Figure 3A) and in males (Figure 3B) with stronger effects by ANP in both these groups. In the female tissue, however, no significant difference was found between ANP- or CNP-induced cGMP production (Figure 3C). When comparing the Delta_cGMP_(ANP-CNP) between males and females (Figure 3D), no significant differences could be detected either. Also, ANP (Figure 3E) or CNP (Figure 3F) treatment was not different in both sexes. These results agree with the expression of Gc-a and Gc-b (see Figure 2G).

**Figure 4:**
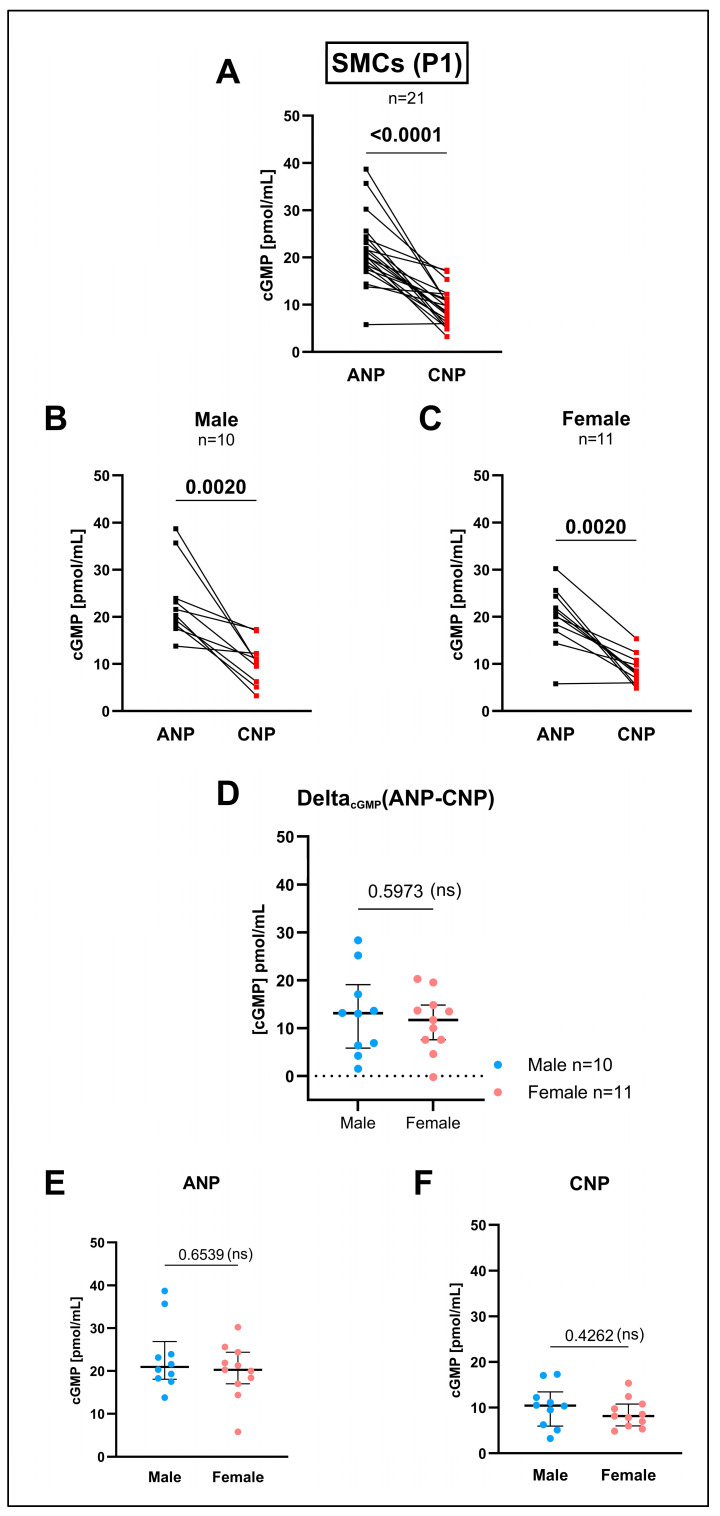
Measurement of cyclic guanosine monophosphate (cGMP) release after the natriuretic peptide (NP) treatment of the cultured aortic smooth muscle cells (SMCs) of the first passage after extraction (P1). The cGMP concentrations of the sample supernatants, given in pmol/mL. cGMP measurement, followed a 30 min incubation time with either atrial natriuretic peptide (ANP) or C-type natriuretic peptide (CNP) at a concentration of 100 nm each. The basic cGMP level of the IBMX (phosphodiesterase inhibitor) control treatment was on average 0.48 pmol/mL. (**A**–**C**) Lines connect the single values for the ANP- (black) and CNP- (red) stimulated cGMP of the cells extracted from the same individuum. Differences in the ANP- and CNP-induced cGMP release in (**A**) the total study cohort (n = 21), and (**B**) in the male (n = 10) and (**C**) female rats (n = 11) only were calculated by two-tailed non-parametric Wilcoxon’s matched-pairs signed rank test. (**D**) To explore the influence of sex on the cGMP release, the difference between the ANP- and CNP-induced cGMP release was calculated as Delta_cGMP_(ANP-CNP) and compared between the male (light blue dots) and female (pink dots) individuals by non-parametric Mann–Whitney test of unpaired measurements. Additionally, the (**E**) ANP- and (**F**) CNP-induced cGMP release between the male (n = 10) and female rats (n = 11) was calculated by non-parametric Mann–Whitney test of unpaired measurements. Individual values are depicted for each gene. The median is indicated by a thick line while whiskers indicate the interquartile range. Numeric *p*-values are given for each comparison while significant differences are highlighted in bold and non-significant differences are indicated with (ns). Due to the reduced n-number in the male and female groups compared to the total cohort, the resulting *p*-values should be interpreted in an explorative manner.

Also, in the cultured SMCs (first passage after extraction), ANP-induced cGMP production was significantly higher than the cGMP levels after the CNP treatment (Figure 4A). This was unexpected when recalling the inverse expression levels for Gc-a and Gc-b of exactly the same cells (Gc-a < Gc-b, see Figure 1B). Surprisingly, this time, even the female cohort (Figure 4C) showed a significantly higher cGMP production after the ANP treatment, and not only the total (Figure 4A) and male group (Figure 4B), in contrast to the findings in intact media (see Figure 3A–C). Again, no sex differences could be detected neither for the ANP- nor CNP-induced cGMP production (Figure 4D–F).

**Figure 5:**
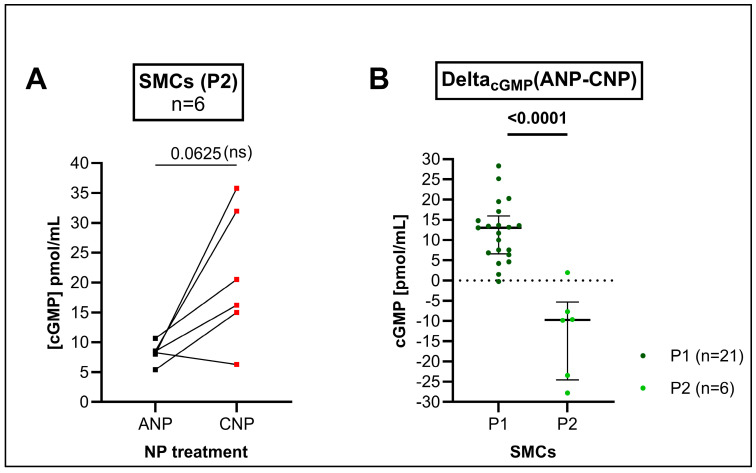
(**A**) Measurement of cGMP release after the natriuretic peptide (NP) treatment of the cultured aortic smooth muscle cells (SMCs) of passage 2 (P2). The cGMP concentrations of the sample supernatants given in pmol/mL. The cGMP measurement followed a 30 min incubation time with either ANP or CNP at a concentration of 100 nm each. The basic cGMP level of the IBMX (phosphodiesterase inhibitor) control treatment was on average 0.09 pmol/mL. Lines connect the single values for atrial natriuretic peptide (ANP)- (black) and C-type natriuretic peptide (CNP)- (red) stimulated cGMP of the cells extracted from the same individuum. Differences between the ANP- and CNP-induced cGMP release in the total study cohort (n = 6) were analyzed by two-tailed non-parametric Wilcoxon’s matched-pairs signed rank test. The numeric *p*-value was found to be not significant (ns). (**B**) Comparison of Delta_cGMP_(ANP-CNP) between the cells of the first (P1, n = 21) and second passage (P2, n = 6) by unpaired non-parametric Mann–Whitney test. Due to the reduced n-number of the P2 cohort, the resulting *p*-value should be interpreted in an explorative manner.

We could not detect a significant difference in cGMP release in response to ANP or CNP in P2 SMCs (Figure 5A). This was in contrast to P1 where ANP induced a higher cGMP release (see Figure 4A). However, the Delta_cGMP_(ANP-CNP) was found to be significantly different when comparing P1 with P2 (Figure 5B).

## 4. Discussion

The investigated genes GC-B, NPRC, and sGC showed a dramatic increase in transcription in the cultured primary SMCs when compared to the corresponding intact aortic media (see Figure 1). The mRNA level of GC-A, however, remained stable throughout cell extraction and culturing.

In recent decades, isolated and cultured SMCs have been used regularly to characterize the expression and function of SMCs in intact tissues including vasculature. The stable expression of *Gc-a* was in contrast to early studies by Suga and colleagues [26,27]. They suggested a downregulation of *Gc-a* expression in primary rat SMC culture. Their conclusions mostly relied on Northern blot analysis using the common housekeeping gene beta-actin. We previously tested several RGs by qPCR [15], amongst them, classical housekeeping genes such as beta-actin, and found that the expression of beta-actin differed dramatically between the intact aortic media and cultured primary aortic SMCs. The difference in the *Gc-a* expression between Suga et al. and our study could be due to the use of beta-actin as RG. When using the RG U2 [22] previously validated for the comparison of intact aortic tissue with its corresponding primary SMCs [15], it was surprising to see that *Gc-b* but not *Gc-a* expression significantly changed upon culturing. Since both receptors not only share their localization within SMCs [28,29] but also structural and regulatory features [11,28,30], a differing role of both cGMP-producing NPRs in cultured SMCs in comparison to intact tissue can be assumed, most likely due to a switch in the SMC phenotype [31,32]. Furthermore, it could be concluded that *Gc-a*, with its stable expression, is the only gene investigated that was found at the “physiological” (tissue) level. It should be mentioned that this does not necessarily imply that GC-A fulfills the same functions in the cultured cells as it does in the intact tissue.

However, when defining an RG, a gene expression unimpaired by experimental conditions is crucial [33]. Regardless of its biological function, this could indicate the suitability of *Gc-a* as a potential housekeeping gene for the comparison of intact vascular tissue and cultured SMCs.

The gene that stood out by the most impressive increase in expression upon culturing of all the genes investigated in this study was the NP clearance receptor Nprc [12]. The reasons for the increase in *Nprc* expression remain speculative. First and foremost, it is likely that an increase in NP clearance counteracts the excessive activity of cGMP signaling by NPs in cell culture. The simultaneous increase in *Gc-b* (and *sGC*), however, suggests further NPRC functions in cell culture. It has been reported earlier that the functions of NPRC reach beyond that of the mere NP clearance [13,14,34]. One function could be the cellular response to oxidative stress which was found to be attenuated by enhanced NPRC expression [35]. In addition, the anti-proliferative capacities of NPRC were suggested for vascular SMCs [36]; an increase in *Nprc* expression, thus, could be the response to a boost in proliferation or an increased oxidative stress, both evident in cultured vascular SMCs [37]. Since the increase in *Nprc* expression seems to happen upon culturing, *Nprc* may be considered as a potential marker gene to identify the synthetic phenotype of vascular SMCs.

Besides the receptors for the NPs, we decided to additionally investigate the expression of *sGC*, the receptor for NO-induced vascular SMC relaxation [8,9,38]. It is of interest that the cytosolic cGMP-producing *sGC* also shows an increased expression (see Figure 1F) like the NP receptors *Gc-b* and *Nprc* upon the culturing of the vascular SMCs.

Awareness of sex differences become increasingly recognized in the field of cardiovascular research. Male and female patients show different symptoms associated with certain diseases [39,40,41] or show different responses to treatment and medication [16,42]. Also, in vitro studies have shown that the two sex hormones (testosterone versus estrogen) may influence cultured vascular SMCs in different ways [18,42,43]. This increasing awareness regarding the potential influence of the biological sex led us to include an equal number of both male and female study subjects to investigate the NP/cGMP cascade.

When comparing the male and female cohort, their expression profiles seemed very similar (see Figure 2G,H); however, a few differences in the sexes were noticeable. Only in the female primary SMCs, *Nprc* significantly exceeded the expression of *Gc-b* and *sGC* (see Figure 2C,D) correlating to a much higher mean value of NPRC expression in the female cohort. However, when comparing *Nprc* expression between males and females directly (see Figure 2H), no significance was found most likely due to a larger spread in the female group. Such larger spreads in the female group were also found for other genes (see Figure 2B,D). Since all the female rats included in this study were of the same strain, age, and nearly the same weight, this variability may be explained by different estrus stages. Thus, it would be most interesting to correlate the level of expression with the hormonal status, determined at the time of organ harvest [44]. Previous studies in the heart have already shown sex-specific differences in ANP-induced cGMP [20].

We determined the enzyme activity of GC-A and GC-B by cGMP ELISA upon ANP or CNP treatment in intact tissue and cultured SMCs. In intact aortic tissue, the cGMP measurements showed a significant difference between the ANP- and CNP-induced cGMP production in the male but not in the female study group (see Figure 3B,C) after the treatment of one half of the aorta with ANP and the other with CNP and using a pairwise analysis. However, when comparing ANP-induced cGMP levels between male and female rats using an unpaired analysis, no significant difference was visible (see Figure 3E). Comparable results were found for the CNP effects (see Figure 3F). The significance in the pairwise analysis might be due to the large spread of the ANP-induced cGMP effects. The reasons for this spread in intact tissues, normalized to the tissue weight, are not yet understood. Similar significant effects were found in the cultured SMCs (first passage after extraction) when comparing sex-specific ANP- and CNP-induced cGMP production using pairwise analyses (see Figure 4B,C) in contrast to ANP- or CNP-effects separately with non-pairwise analysis (see Figure 4E,F).

In the SMCs of passage 1 (P1), ANP treatment induced higher cGMP levels than CNP (see Figure 4A). This was inverse to the mRNA expression of the ANP and CNP receptors in the SMCs of the same passage and from the same animals, i.e., *Gc-b* significantly exceeded *Gc-a* expression (see Figure 1B). In the following passages (P2, P3), however, the CNP-induced cGMP production of the cultured cells began to exceed the ANP-induced cGMP production. Even though this effect was readily visible in graphs (Figure 5), it did not reach statistical significance when performing pairwise comparisons. This might reflect that the typical *Gc-a* and *Gc-b* pattern at the mRNA level (*Gc-b* mRNA > *Gc-a* mRNA) in cell culture has now been translated to the protein level (GC-B > GC-A) visible by the receptors’ cGMP production.

Our data illustrate that NP receptor expression seems to rapidly adopt a new balance upon cell isolation with subsequent changes in receptor functionality. With the remarkable increase in *Nprc* expression, the local control of NP availability is affected and might change regular NP-induced cGMP signals. These disrupted signals in cell culture may obscure physiological mechanisms and lead to the misjudgment of the entire pathway impeding, e.g., drug discovery.

In this regard, it is interesting that cultured vascular SMCs share characteristics with SMCs in the walls of diseased blood vessels [31,45]. Thus, it would be interesting to see whether a similar expression profile is found in the tissue of aortic pathology compared to SMC culture. Cultured vascular SMCs may potentially serve as an in vitro disease model in the future [1,46].

Given that the isolation of cells rapidly induces changes at the transcription level, RNAseq data should be interpreted with caution. Emerging techniques like spatially resolved transcriptomics using intact tissue slices may provide a more realistic view of the protein expression profile and hold the potential to unveil new yet unknown clinically relevant biomarkers that may be missed in single cell-based approaches.

## 5. Conclusions

Regarding NP-dependent cGMP signaling, profound changes in the expressional and functional landscape could be shown in response to the mere cell isolation of vascular (aortic) SMCs. The drastic increase in *Nprc* expression most certainly distorts NP-dependent cGMP signals in isolated and cultured cells. Data illustrate that cell-based studies carry an inherent risk of misjudgment and even challenge modern single-cell-based approaches such as RNAseq.

## Figures and Tables

**Table 1:** Cycler steps for RT-qPCR.

	Step	Cycles	Duration	Temperature
1	Polymerase activation	1×	3 min	95 °C
2	Denaturation	35×	20 s	95 °C
3	Primer hybridization		20 s	60 °C
4	Elongation		20 s	72 °C
5	Melt curve analysis			55–95 °C

## Data Availability

The data that support the findings of this study are available from the corresponding author upon reasonable request.

## Abbreviations

ANP, atrial natriuretic peptide; BNP, B-type natriuretic peptide; cGMP, cyclic guanosine monophosphate; CNP, C-type natriuretic peptide; GC, guanylyl cyclase; GC-A, guanylyl cyclase A; GC-B, guanylyl cyclase B; NO, nitric oxide; NP, natriuretic peptide; NPRC, natriuretic peptide clearance receptor; P, cell culture passage; PCR, polymerase chain reaction; qPCR, quantitative real-time PCR; RG, reference gene; RT, reverse transcriptase; sGC, soluble guanylyl cyclase; SMC, smooth muscle cell; U2, small nuclear ribonucleoprotein 2.
